# Bidirectional regulation of lipid metabolism and the tumor microenvironment: new perspectives from mechanism to therapy

**DOI:** 10.3389/fimmu.2025.1696102

**Published:** 2025-11-11

**Authors:** Kaixin Shi, Bing Pan, Ruixin Zheng, Kaiyi Liu, Jincheng Song, Xiaobo Wang, Li Li

**Affiliations:** 1 Department of Hematology, The Second Hospital of Dalian Medical University, Dalian, China; 2 Department of Oncology, The Second Hospital of Dalian Medical University, Dalian, China

**Keywords:** lipid, lipid metabolism, metabolic reprogramming, cancer, tumor microenvironment, immune response

## Abstract

The role of lipid metabolism in cancer and immune regulation has received significant attention in recent years. Reprogramming of lipid metabolism is one of the key hallmarks of cancer and plays a critical role in cancer progression by supporting the rapid proliferation, survival, and metastasis of tumor cells. Importantly, beyond its well-established functions in cancer cells, lipid metabolism dynamically regulates the functions of various immune cells within the TME (e.g., T cells, natural killer cells, macrophages), thereby molding antitumor immune responses. This review combines the contemporary awareness of the reciprocal interactions between lipid metabolism and the TME. We start with a simple overview of key lipid metabolic pathways in cancer cells, followed by an in-depth exploration of the way lipid uptake, synthesis, and oxidation influence the fate and role of tumor-infiltrating immune. We also appraise the translational potential of targeting lipid metabolism and propose that combining inhibitors of key metabolic enzymes, for example fatty acid synthase or acetyl-CoA carboxylase, with immunotherapy can not only effectively alleviate immunosuppression but also overcome immunosuppression. Finally, we spotlight the remaining knowledge gaps and put forward future research priorities and potential. Intervening in lipid metabolic interactions represents a promising prospect for developing the novel cancer treatment strategies.

## 1 Introduction

Cancer is the second most common cause of death worldwide and is projected to be the leading cause of death by 2060 (1). In recent years, there has been a significant increase in the interest of abnormal metabolism in cancer cells, and an increased focus on the increasingly important role of lipids in tumorigenesis and cancer progression. In order to survive and divide under unfavorable conditions, such as hypoxia and nutrient-poor conditions, cancer cells re-model the surrounding immune and stromal cells, and consequently build a supportive TME. Within this TME, multiple components interact to provide energy for the tumor and regulate tumor progression through multiple signaling pathways (2). Cancer cells exhibit uncontrolled proliferation and have a high demand for energy. Although angiogenesis is enhanced in the TME, the tumor vasculature cannot fully provide the necessary demands of cancer cells. Therefore, cancer cells reprogram their metabolism and hyperactivate lipid metabolism to support their proliferation.

Growing evidence has revealed that lipid metabolism is generally enhanced at multiple stages of cancer development. Cancer cells contain elevated lipid levels, such as increased uptake of exogenous lipids and lipoproteins, as well as over-activated *de novo* lipid synthesis. These events directly promote the malignant transformation and progression of tumor cells, as well as the accumulation of abnormal lipids in the TME (3). For instance, the expression of fatty acid synthase (FASN) is upregulated in TNBC, causing lipid accumulation. In addition, lipid metabolism also provides energy for tumor cells. For example, the products of (FAO), such as reduced nicotinamide adenine dinucleotide, flavin adenine dinucleotide, reduced nicotinamide adenine dinucleotide phosphate and adenosine triphosphate (ATP), are important energy sources for tumor cells under hypoxic conditions (4).

In the past few years, lipids have been shown not only as an alternative energy source to bridge energy deficits but also as essential components in the synthesis of biomembranes, substrates for biomass and as activators of highly complex signaling pathways regulating cancer cell growth and migration (5). Such characteristics have been described in several types of cancer such as breast, colorectal and ovarian cancer and are highly correlated with a poor prognosis (6). In addition, lipid metabolism also influences the function of immune cells within the TME, which interact with cancer cells to form a complex network of metabolic crosstalk that critically regulate anti-tumor immunity and ultimately determine tumor progression. There are considerable clinical gaps in translating the understanding of these mechanistic insights into therapies. One of the main challenges is the heterogeneity of lipid metabolic dependencies between patients and cancer types, which limits the identification of universal therapies. The other major challenge is that the regulation between lipid metabolism and function of immune cells is a poorly studied mechanism for the development of new therapies, especially for the overcoming resistance to conventional chemotherapy and immunotherapy. In this review, we perform an in-depth discussion of the mechanistic interplay between lipid metabolic reprogramming in cancer cells and the immune microenvironment of the TME. We critically discuss how the modulation of these highly complicated interconnected pathways represents a promising therapeutic strategy to overcome treatment resistance and improve cancer outcomes. By discussing the interplay between cancer cells and the TME, we fill the existing clinical gaps and uncover unique mechanistic insights that provide novel research directions for cancer immunotherapy. We provide a solid theoretical basis for developing personalized metabolic therapies by determining key metabolic nodes that can be exploited based on the specific tumor and immune context. These new findings reveal the translational potential of targeting lipid metabolism to overcome therapeutic resistance and improve prognostic outcomes in cancer patients. Thus, providing a new perspective for clinical application.

## 2 Lipid metabolism

**Figure 1:**
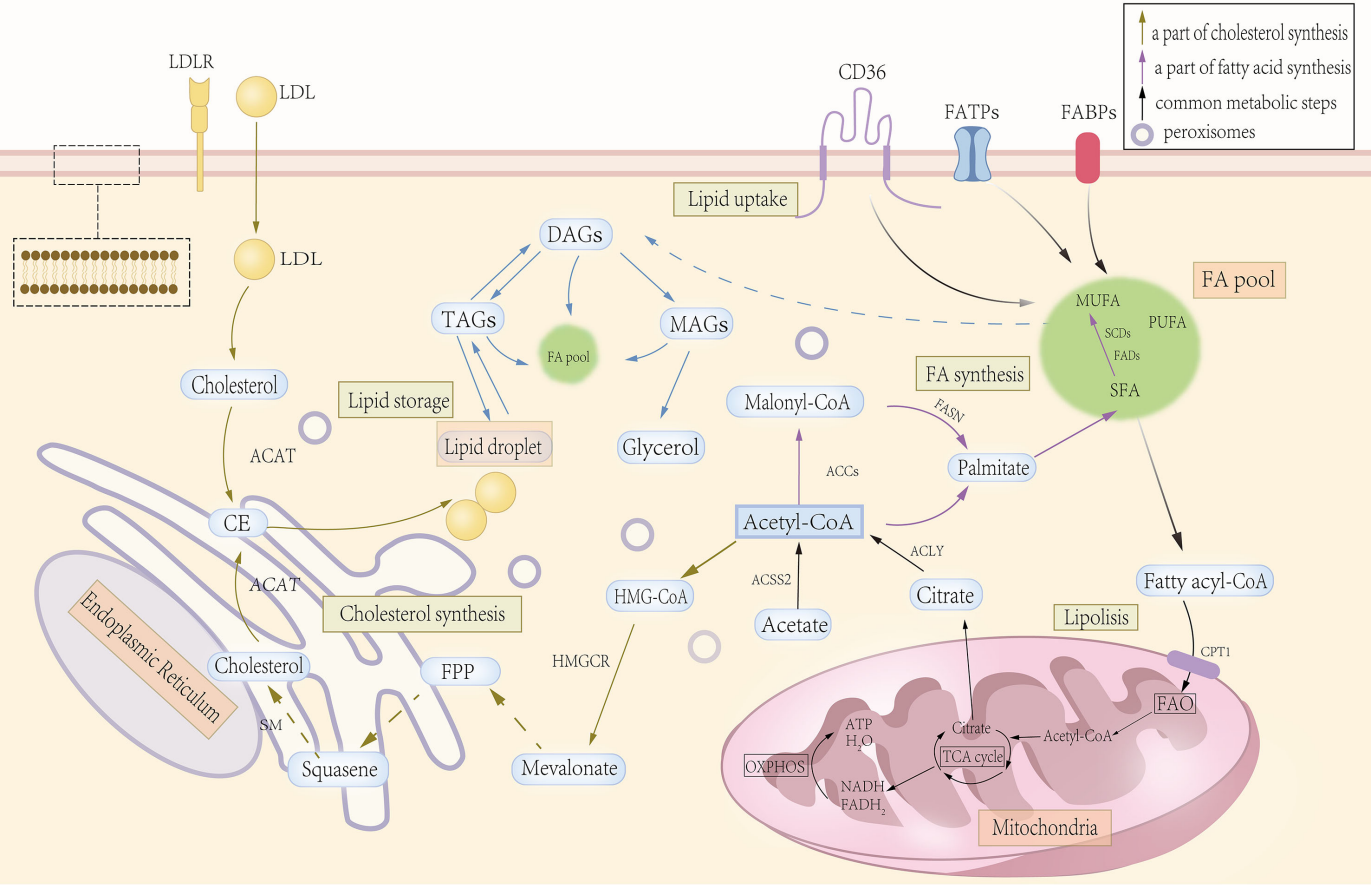
Significant steps in lipid metabolism. Lipid absorption is mainly accomplished through specific channels and transport mechanisms in the intestinal epithelial cells. Lipid synthesis begins with acetyl-CoA, which gradually extends the carbon chain through the fatty acid synthase complex to form fatty acids, which are then esterified with glycerol-3-phosphate to form triglycerides or phospholipids, while cholesterol is synthesized from acetyl-CoA via the mevalonate pathway. Lipid catabolism and oxidation begin with the hydrolysis of triglycerides in adipose tissue to glycerol and free fatty acids by lipase, and fatty acids enter the cell and are catabolized in the mitochondria through β-oxidation to acetyl-CoA, which enters the tricarboxylic acid cycle for complete oxidative energy supply. Inside the cell, lipids (e.g., triglycerides and cholesterol esters) are synthesized on the endoplasmic reticulum (ER). As lipids continue to accumulate, they eventually form separate lipid droplets, which are used for energy storage as well as to regulate lipid metabolism and signal transduction.

Lipid metabolism encompasses the processes of lipid synthesis, catabolism, transport, and regulation (7). The regulation of lipid metabolism maintains cellular homeostasis. In the interest of understanding the metabolic communications of the TME, we summarize the most salient alterations of lipid metabolism in cancer cells (**Figure 1**).

### 2.1 Fatty acids in cancer

#### 2.1.1 Uptake

Cancer cells increase the uptake of exogenous lipids by upregulating the expression of FA transporters, such as CD36, and FABPs (FA-binding proteins) (8). High CD36 expression has been correlated with poor prognosis in breast, ovarian, gastric, and prostate cancer patients (9). Interestingly, different FABP isoforms have opposing functions: A-FABP drives the growth of breast cancer, while E-FABP enhances anti-tumor immunity (10, 11). However, the strong functional antagonism between pro-tumorigenic A-FABP and anti-tumorigenic E-FABP poses a major obstacle in the translational application of these two isoforms, calling for highly specific targeting strategies. The first challenge that the TME poses to metabolic oncology is also the biggest, namely, how does the TME decide which FABP isoform wins the battle? Future studies should focus on identifying the upstream signals and downstream effectors that confer this context-dependency to avoid further immunosuppressive or tumor-promoting effects from the TME. This challenge also offers a promising opportunity in metabolic oncology.

#### 2.1.2 Synthesis

*De novo* lipogenesis is the most important source of fatty acids (FAs). Studies indicate that approximately 95% of FAs are synthesized endogenously by cancer cells despite the availability of extracellular FAs. Its principal substrate, acetyl-CoA, is produced from citrate by ATP citrate lyase (ACLY) or from acetate by acetyl-CoA synthetase (ACSS2).

##### 2.1.2.1 Acetyl-CoA-producing enzymes: ACLY and ACSS

Expression or activity of ACLY is associated with tumor progression in glioblastoma, colorectal, breast, lung, and hepatocellular carcinomas and promotes cancer cell proliferation and stemness. Consequently, ACLY has emerged as a promising therapeutic target. However, ACLY inhibition induced immunosuppression in immunocompetent mice by promoting the production of immunosuppressive polyunsaturated fatty acid peroxidation products, activating the cGAS-STING pathway, and upregulating PD-L1, which drives immune evasion and metastasis (12). In human cancers, reduced ACLY expression correlates with cGAS-STING activation and decreased T-cell infiltration. Therefore, since ACLY inhibition may carry an immunosuppressive effect, ACLY inhibitor monotherapy for cancers in preclinical models and clinical trials needs careful consideration and evaluation (12). This example clearly demonstrates that one cannot think of attacking a key metabolic enzyme in isolation of its dramatic impact on the immune landscape and renders it a “combination” approach from the beginning. Obviously, the anti-PD-1/PD-L1 combination therapy could have blocked this immunosuppressive signal and enabled the activated T cells to mediate their cytotoxic activities and thus, overcame the immunosuppression emerged from ACLY inhibitor treatment to obtain a synergistic antitumor effect. This suggests that inhibition of ACLY with immune checkpoint blockage may be a sustained anticancer strategy.

Acetyl-CoA synthetase (ACSS): ACSS1 and ACSS3 are expressed in mitochondria, whereas ACSS2 is expressed in the cytoplasm and nucleus (13). Previous studies have concentrated on ACSS2, which is upregulated in cancer cells under stress conditions, such as nutrient deprivation and hypoxia. Depletion/reduction of ACSS2 has been reported to impair the growth of multiple cancer types, including breast, prostate, liver and glioblastomas (13–15). This highlights the critical importance of ACSS2 in tumor growth and thus justifies its use as an anticancer agent. In addition, ACSS2 is involved in promoting the use of acetic acid for lipid synthesis and the growth of tumors (14, 16). ACSS2 deletion also protects cells and mice from fibrosis development, suggesting its potential as a therapeutic target for renal diseases (17, 18). Knockdown of ACSS1 reduces hepatocellular carcinoma cell viability and suppresses melanoma tumor growth (18–20). ACSS3 inhibits prostate cancer progression by increasing endoplasmic reticulum stress-mediated apoptosis through downregulation of the lipid droplet-associated protein PLIN3 and reverses drug resistance by reducing lipid droplet deposition in tumors (21).

##### 2.1.2.2 FA biosynthetic enzymes: ACC, FASN, and SCD

Acetyl-CoA carboxylase (ACC): Mammalian ACC has two tissue-specific isoforms: ACC1 and ACC2. ACC1 is highly expressed in various human cancers, including breast, gastric, liver, and prostate cancers, correlating with reduced patient survival (22, 23). ACC2 is highly expressed in laryngeal cancer, and its expression levels are positively correlated with advanced clinical stages and reduced 5-year survival (24). Under nutrient-rich conditions, prolyl hydroxylase domain protein 3 hydroxylates and activates ACC2, inhibiting FAO. In acute myeloid leukemia (AML), reduced prolyl hydroxylase domain protein 3 expression promotes FAO, driving AML cell proliferation and disease progression (25).

Fatty acid synthase (FASN): FASN expression is upregulated in early-stage lung, prostate, and breast cancers (26–28), with further increases observed as cancer progresses (29, 30). This indicates that cancer cells express enhanced *de novo* lipid synthesis at an early stage of tumorigenesis and is associated with cancer recurrence and poor survival (31). Inhibition of FASN can decrease FA synthesis, increase the accumulation of malonyl-CoA and inhibit CPT1-mediated FAO, induce cell cycle arrest and apoptosis (31, 32). Moreover, FASN inhibitors can block palmitoylation and plasma membrane- and mitochondria-associated EGFR activation and enhance cancer cell sensitivity to EGFR inhibitors by promoting EGFR ubiquitination, which further results in attenuating tumor growth (33, 34).

Stearoyl-CoA desaturase (SCD): SCD catalyzes the conversion of saturated fatty acids (SFAs) to monounsaturated fatty acids (MUFAs) and converts stearoyl-CoA (C18:0) and palmitoyl-CoA (C16:0) to oleoyl-CoA (C18:1) and palmitoyl-CoA (C16:1), respectively, with the introduction of double bonds, which increase the level of FAs unsaturation and serve as an important energy source for tumor cells. SCD is highly expressed in several human cancers, such as lung, breast, colorectal, renal, prostate and hepatocellular carcinomas (35–37), and is associated with poor survival (38). Downregulating SCD expression can induce cell cycle arrest and apoptosis and inhibit the proliferation of lung cancer cells (39). SCD inhibitors are particularly effective in blocking cancer cell proliferation, as their effects are linked to AMPK activation, which phosphorylates ACC and inhibits FA synthesis (40). This prevents excessive saturated FA accumulation while blocking desaturation.

#### 2.1.3 Catabolism and FAO

It has been demonstrated that lipolysis plays a critical role in lipid-mediated signaling processes. Lipids released from low-density lipoprotein (LDL), such as certain FAs, are not only vital sources of energy but also signaling molecules, directly regulate cellular signaling pathways and gene transcription. For example, FAs or FA derivatives can bind to and activate members of the nuclear receptor family of transcription factors, such as peroxisome proliferator-activated receptors (PPARs), which regulate gene expression and influence lipid metabolism and inflammatory genes (41).

**Figure 2:**
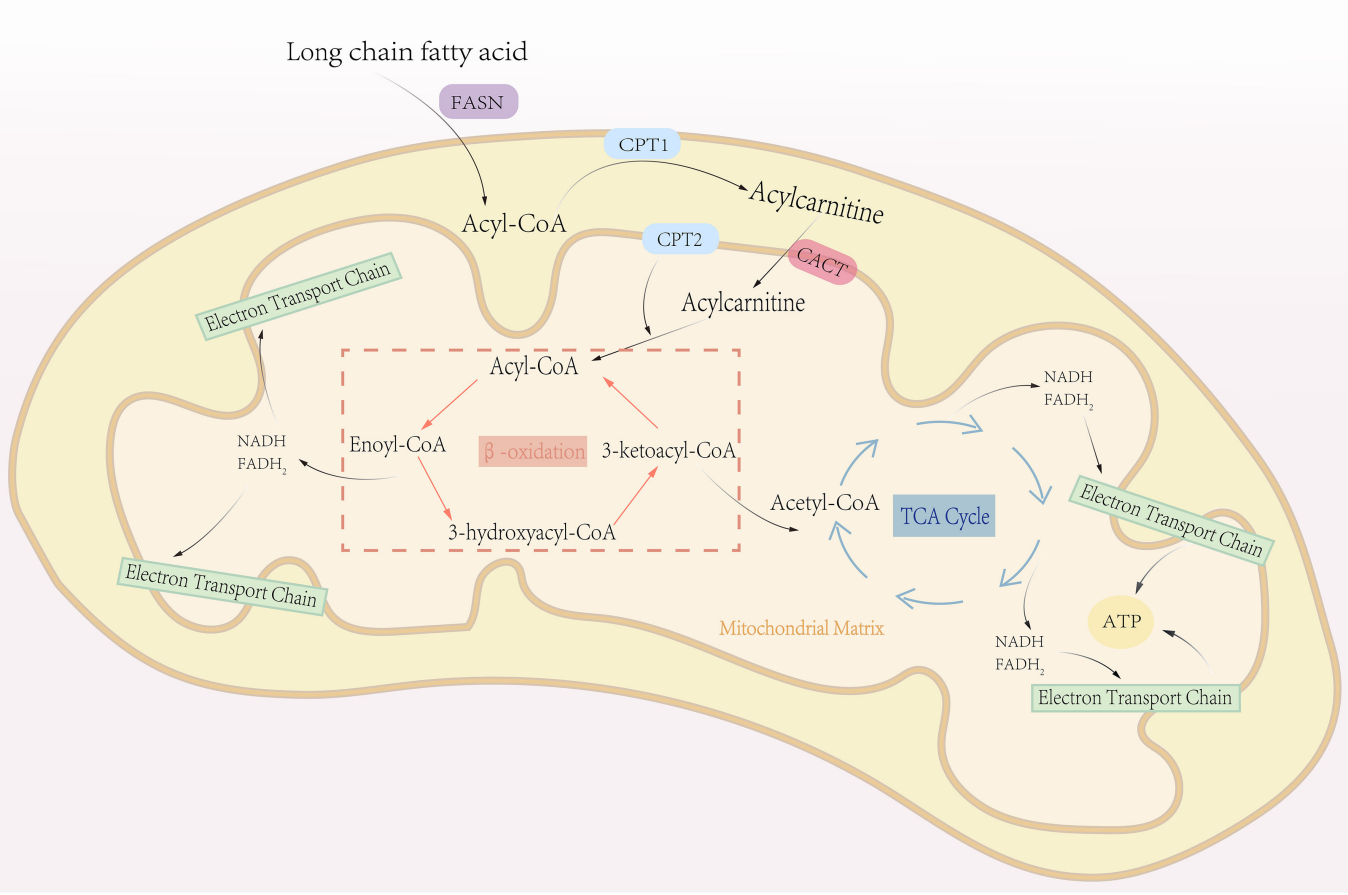
The process of fatty acid oxidation. Fatty acid oxidation, also known as β-oxidation, is a central metabolic pathway that breaks down fatty acids to generate energy. This process primarily occurs in the mitochondrial matrix. Triglycerides stored in adipose tissue are hydrolyzed into glycerol and free fatty acids, which are then activated by combining with CoA to form fatty acyl-CoA. Fatty acyl-CoA is transported into the mitochondrial matrix via the carnitine shuttle system. Within the mitochondrial matrix, fatty acyl-CoA undergoes β-oxidation, a cyclic process that sequentially removes two-carbon units in the form of acetyl-CoA. Each cycle of β-oxidation generates one molecule of NADH and one molecule of FADH_2_. The acetyl-CoA produced enters the TCA cycle, where it is fully oxidized to produce ATP. Simultaneously, NADH and FADH_2_ donate electrons to the electron transport chain, driving oxidative phosphorylation to generate additional ATP. This process repeats iteratively until the fatty acid is completely broken down into acetyl-CoA.

FAO plays a critical role in cell proliferation and is closely associated with ATP production and nicotinamide adenine dinucleotide homeostasis, both of which are essential for mitochondrial function and cell survival (**Figure 2**). High FAO activity has been reported in several cancers, such as triple-negative breast cancer and gliomas (4, 42). I In glioblastoma cells, the inhibition of FAO leads to a decrease in the levels of NADPH and an increase in ROS levels, which promote apoptosis (43). However, FAO has dual functions: it maintains cell survival under stress conditions but promotes cytotoxicity when excessive. This suggests the need for strict regulation of cancer cells and highlights an attractive target for cancer therapy because strict regulation rather than complete inhibition of FAO is required. Thus, maintaining a balance between the production of ATP and nicotinamide adenine dinucleotide phosphate through FAO inhibition prevents lipotoxicity, and dynamic regulation of FAO can inhibit the proliferation and survival of tumor cells. Consistently, key enzymes and transporters involved in FAO are often upregulated in various human cancers. In breast cancer, mammary adipocyte-derived leptin upregulates STAT3 and induces the expression of CPT1B and enhances FAO activity in cancer stem cells, promoting stemness and chemotherapeutic resistance (44). The transcriptional regulators, such as PPARs and AMP-activated protein kinase, can activate the expression of transcriptional regulators to promote FAO (45).

#### 2.1.4 Storage

Lipid droplets (LDs) are important organelles involved in lipid and energy storage. It is well accepted that LDs are derived from endoplasmic reticulum (46). LDs are involved in energy storage and serve as the main source of precursors for cell membrane phospholipids and cholesterol (47). LDs have a dynamic life cycle in cells and their size, intracellular location, and lipid and protein composition can change rapidly in response to various environmental conditions and cell states (48). Enzymes and proteins involved in lipid metabolism localize to the surface of LDs and regulate lipid homeostasis by maintaining a balance between lipid storage and catabolism (49). LDs also promote lipid catabolism through the action of neutral lipases, such as ATGL and HSL. These enzymes mediate the breakdown of triglycerides to release large amounts of free FAs that can be transported to mitochondria for oxidation to produce ATP to meet cell energy demands (50, 51).

### 2.2 Cholesterol in cancer

Cholesterol participates in the composition of biological membranes, maintaining the fluidity of lipid bilayers, forming lipid rafts, and solubilizing other lipids to coordinate the activation of many signal transduction pathways Altered cholesterol metabolism facilitates the proliferation of tumor cells and induces the apoptosis of tumor cells by regulating the oncogenic pathway PI3K/AKT/mTOR, and it induces CD8+ T cell exhaustion to create an immunosuppressive TME (52–54). Cholesterol is regulated by two pathways: dietary intake and *de novo* synthesis through the mevalonate pathway. Transcription factors involved in the regulation of these pathways include SREBPs and LXRs.

#### 2.2.1 Uptake

Cancer cells have high demands for cholesterol and enhance uptake to meet these demands by overexpressing LDLR. This has been demonstrated in hepatocellular, breast, and prostate cancers, and high expression of LDLR correlates with poor prognosis (55–57). Deletion of LDLR prevents the growth of tumors in HER2+ breast cancer and PDAC models hyperlipidemic mice (58). In prostate cancer cells, deletion of PTEN leads to the activation of PI3K/AKT, and this greatly enhances the uptake of exogenous LDL; this process is essential for the growth of tumors (59). Abnormalities in lipid metabolism can cause lipotoxicity, inducing oxidative stress and causing a large increase in ROS. Progressive oxidative stress can induce the oxidative modification of intracellular LDL to ox-LDL. Ox-LDL can bind to scavenger receptors, such as LOX-1, an oxidized LDL receptor-1, and CD36 (60), causing mutations that induce inflammation, cell proliferation, and metastasis. Based on the experimental evidence above, empirical studies have demonstrated the pro-tumorigenic effects of these molecules. Zettler et al. reported that ox-LDL upregulates cyclin expression, which leads to an increase in cell proliferation (61). Similarly, another study reported that treating ovarian cancer cells with ox-LDL enhances their proliferative capacity (62). Furthermore, oxidative stress can accelerate DNA damage in cancer cells, which leads to the malignant transformation of cells and carcinogenesis (63).

Due to the complexity of the mechanisms underlying the role of LDL and its oxidized forms in cancer, little is known about how LDL, through its receptors, regulates the reprogramming of lipid metabolism in cancer cells, and how it activates signaling pathways within cancer cells through their receptors, leading to oxidative stress and inflammatory responses, and how it regulates the function of immune cells, which is important for exploring its potential as a target for inducing oxidative stress and inflammatory responses, and for developing novel therapeutic interventions or combined treatment regimens, which is crucial for fully exploring its potential as a target.

#### 2.2.2 Synthesis

##### 2.2.2.1 Cholesterol biosynthetic enzymes: HMGCR and SM

3-Hydroxy-3-methylglutaryl-CoA reductase (HMGCR). HMGCR is the rate-limiting enzyme in cholesterol biosynthesis, overexpressed in gastric, glioblastoma and prostate cancers, and its upregulation facilitates cancer cell growth and migration, while its knockdown attenuates tumorigenesis. Therefore, HMGCR inhibition may provide a promising therapeutic approach for solid tumors, hematologic malignancies and drug-resistant cancers (64–66). However, the risk reduction associated with the two polymorphisms might be affected by confounding factors or biases inherent to observational studies, which limit the possibility of establishing a causal relationship. Therefore, further mechanistic studies and prospective clinical trials are required to confirm whether HMGCR inhibition per se confers a direct anti-cancer effect or is only associated with a lower underlying risk.

Squalene monooxygenase (SM). SM is the second rate-limiting enzyme in cholesterol biosynthesis, located downstream of HMGCR, and is also regulated by SREBP2 (67). Its stability is regulated by its substrate and lipid interactions: squalene prevents its ubiquitination and subsequent degradation by MARCH6, while unsaturated FAs inhibit its degradation (68, 69). Defective SM expression was observed in anaplastic lymphoma kinase (ALK)-positive anaplastic large-cell lymphoma cells, leading to cholesterol dysregulation and dependence of tumor growth on LDL receptor (LDLR)-mediated cholesterol uptake (70).

#### 2.2.3 Efflux and storage

ATP-binding cassette (ABC) transporters, including ABCA1 and ABCG1, mediate cholesterol efflux to maintain homeostasis. In tumor-associated macrophages (TAMs), ABCA1/ABCG1-driven efflux potentiates IL-4/STAT6 signaling, enforcing an M2-like, pro-tumorigenic phenotype. Genetic ablation of these transporters reverses TAM-mediated immunosuppression and impairs angiogenesis (71, 72). This positions cholesterol efflux inhibition as a promising strategy to reprogram TAMs. LXRs and their downstream target genes, such as the ABC family, enzymes involved in lipid metabolism, and extracellular lipid receptors, regulate cholesterol transport and downregulate genes related to lipid uptake and synthesis. These mechanisms may contribute to the formation of an immunosuppressive TME. LXRs promote lipogenesis and the transcription of ABCA1 and ABCG1 (73). To prevent cytotoxicity from excessive free cholesterol accumulation, acyl-CoA cholesterol acyltransferase (ACAT) converts cholesterol into cholesteryl esters, which are stored in LDs (74). Targeting this storage mechanism may offer another avenue to disrupt cholesterol homeostasis in cancer cells.

## 3 Crosstalk between lipid metabolism and tumor immune response in the TME

**Figure 3:**
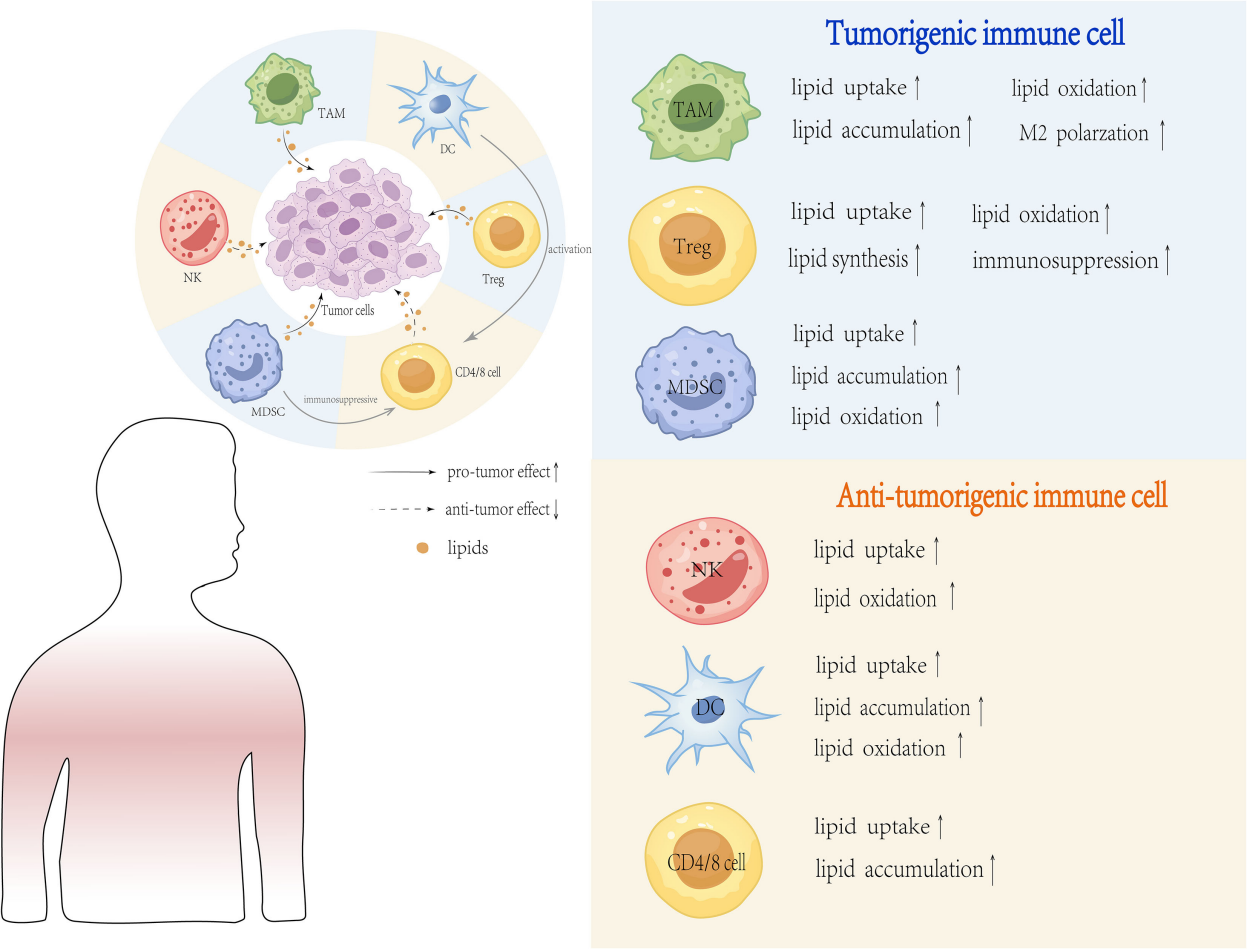
Major Lipid Metabolic Changes in the Tumor Microenvironment. Lipid metabolic changes in the TME are a hallmark of tumor cells adapting to rapid proliferation and survival. These changes include increased lipid uptake and synthesis, lipid storage and utilization, enhanced lipid oxidation, reprogramming of lipid metabolism, lipid-mediated signaling and lipid metabolism in tumor-associated immune cells. These metabolic adaptations enable tumor cells and immune cells to thrive in the harsh TME, facilitating tumor progression and immune evasion.

Despite the harsh conditions of the TME established by tumor cells, immune cells have adapted by utilizing lipids as fuel to support anti-tumor immune responses. The major immune cells involved in these metabolic cross-talks are T cells, tumor-associated macrophages, dendritic cells, neutrophils, natural killer cells and myeloid derived suppressor cells. The response of immune cells to lipid metabolic changes is not uniformly induced but depends on the involvement of a multitude of factors. Factors such as the specific lipid species, type of immune cell and the dynamic TME can determine whether a pro- or anti-tumor response is elicited. As a result, the interpretations drawn from these findings are often context-dependent and appear contradictory (**Figure 3**). This inherent contextual dependency poses a major challenge to translate these findings into effective therapies and calls for the development of individualized context-dependent strategies targeting lipid metabolism in immunotherapy. In this section, we will critically review how distinct lipid metabolic programs not only nourish but also instruct the polarized functional maturation of various immune cells, generating a metabolic landscape that can either resists or responds to immunotherapy. The inherent context-dependency of these interactions is the main challenge of translating these findings into effective universal therapies.

### 3.1 T cells in tumor immunity

T cells are an essential component of the adaptive immune response that provides protection against pathogens and tumors. Naïve T cells can proliferate and differentiate into effector cells upon antigen and co-stimulatory signals as well as cytokines, which are involved in the process of tumor development and progression. CD8+ T cells are believed to be the effector cells of adaptive anti-tumor immunity that can differentiate into cytotoxic T lymphocytes (CTLs) and eliminate tumor cells in an MHC-dependent manner (75). CD4+ T cells include pro-inflammatory T-helper 1 (Th1) cells, immunosuppressive T-helper 2 (Th2) cells, T-helper 17 (Th17) cells and regulatory T-cells (Tregs) that have pro- or anti-inflammatory effects on tumors (76).

Immune signaling pathways, such as phosphoinositide 3-kinase (PI3K)-Akt and mechanistic target of rapamycin (mTOR), regulate T cell lipid metabolism. Elevated levels of cholesterol and FAs in the TME can alter T cell function. T cell receptor (TCR) stimulation activates PI3K-Akt and mTOR signaling, inducing FA and mevalonate synthesis (77, 78). Inhibition of ACC1 reduces Th17 cell differentiation (79) while enhancing memory CD4+ T cell formation (80). ACC1 is also essential for antigen-specific CD8+ T cell accumulation during bacterial infection and Treg suppressive function in the TME (81).

CD36-mediated uptake of lipids, such as long-chain fatty acids and oxidized low-density lipoproteins (ox-LDL), promotes Treg function but can lead to lipid peroxidation, p38-mitogen-activated protein kinase (MAPK) activation, or ferroptosis in CD8+ T cells, impairing their function (82–84). Increased FAs in the TME can activate CD8+ T cells, promoting lipid metabolism and maintaining effector function through peroxisome proliferator-activated receptor alpha (PPARα) signaling (82). Furthermore, enhancing FA catabolism in tumor-infiltrating CD8+ T cells boosts their anti-tumor activity. Lipid depletion in CD8+ T cells significantly impairs their proliferation and cytotoxicity, leading to functional exhaustion characterized by reduced interferon (IFN)-γ production and increased programmed cell death protein-1 (PD-1) expression (85, 86).

Cholesterol and its biosynthetic intermediates also have a profound impact on T cell function in the TME. Cholesterol induces CD8+ T cell exhaustion by inducing endoplasmic reticulum stress and promoting uncontrolled tumor growth (87). LDL, rich in cholesterol, is uptaken by LDL receptor (LDLR). LDL depletion or LDLR deficiency impair CD8+ T cell function and anti-tumor activity (88). These observations provide strong rationale for targeting lipid pathways in T cells.

### 3.2 Tumor-associated macrophages

TAMs are one of the most highly infiltrating immune cells in the TME. TAMs interact with cancer cells and promote tumor growth and progression. Reprogramming of lipid metabolism is a hallmark of TAMs, and lipid metabolites secreted by TAMs promote or inhibit tumor growth. The initial steps of this reprogramming are initiated by a combination of extrinsic cues. These include soluble factors in the TME, such as cytokines (IL-4, IL-13, M-CSF), hypoxia, and uptake of exosomes from tumors. These different signals converge to activate some key transcription factors, including PPARγ and STAT6, and further induce transcriptional upregulation of genes involved in lipid uptake and *de novo* lipogenesis.

TAMs can be further polarized into classically activated (M1) macrophages and alternatively activated (M2) macrophages. M1 macrophages participate in anti-tumor immune responses by secreting pro-inflammatory cytokines, such as tumor necrosis factor-alpha (TNF-α), and inducible nitric oxide synthase (iNOS). In contrast, M2 macrophages are characterized by an anti-inflammatory phenotype and secrete pro-tumor factors, such as arginase 1 (ARG1) (89). Peroxisome proliferator-activated receptors (PPARs) are involved in almost all aspects of FAs metabolism (37) and have been shown to regulate TAM polarization toward the M2 phenotype through multiple signaling pathways, promoting tumor cell proliferation, angiogenesis, and immunosuppression (90, 91). Both PPARγ agonists and inhibitors exhibit anti-tumor activities. PPARγ inhibitors attenuate the pro-inflammatory effects of M1 TAMs and suppress the secretion of pro-tumorigenic cytokines by M2 macrophages, further inhibiting tumor progression (92, 93).

The phenotype of TAMs, expressed as pro- or anti-tumorigenic functions, is directly determined by their underlying lipid metabolic state. Upregulation of critical lipid metabolism genes in TAMs by tumor cells induce lipid accumulation and M2 polarization, which further promote tumor progression.

Reprogramming of cholesterol metabolism regulates TAM activity and recruitment by inducing M2 polarization and tumor progression. Currently, research on cholesterol metabolism in TAMs is focused on the cholesterol efflux pathway. For example, ABCA1/ABCG1-mediated cholesterol efflux is an active driver of M2-like polarization through its enhancement of the IL-4/STAT6 signaling (72). Targeting this pathway to reprogram TAMs appears very attractive; however, this strategy raises a serious safety concern. Systemic inhibition of the reverse cholesterol transport may induce atherosclerosis at the expense of an on-target, off-tumor toxicity. Therefore, safer alternatives are needed for future efforts. We believe future efforts should focus on developing TAM-specific delivery systems or targeting context-specific lipid dependencies (94). Lipid metabolism in TAMs is associated with immunosuppression and chemotherapy resistance. For example, in a gastric cancer mouse model, lipid accumulation in TAMs upregulates the expression of phosphatidylinositol 3-kinase gamma (PI3K-γ), promoting M2 polarization. This decreases phagocytosis and upregulates programmed death ligand 1 (PD-L1) expression in TAMs, attenuating the anti-tumor T-cell response and enhancing immunosuppression (95). TAMs show high plasticity, and targeting lipid metabolism to induce M1 polarization may improve the efficacy of tumor immunotherapy and reduce chemoresistance (96). Considering the important role of lipid metabolism in TAMs, several studies have focused on targeting lipid metabolism in TAMs or inducing their conversion into anti-tumor phenotypes. However, lipid metabolism in TAMs has not been systematically investigated, and little is known about how its initial events are initiated and how it exerts dual roles in cancer. Therefore, further investigation is required to elucidate the mechanisms underlying lipid metabolism in TAMs and to provide a theoretical basis for developing effective therapeutic strategies.

### 3.3 Dendritic cells

DCs and NK cells are critically important for successful anti-tumor immune responses. However, multiple immunosuppressive factors can exist in the TME to impair their function and promote a pro-tumorigenic phenotype (97, 98). DCs are innate immune cells that can be grouped into conventional DCs (cDCs), plasmacytoid DCs (pDCs), and monocyte-derived DCs (moDCs) depending on their respective developmental pathways (99). The main role of DCs is to capture and process antigen, present them to the T lymphocytes, and thus, induce adaptive immune responses. Upon maturation and stimulation of DCs, naïve CD4+ and CD8+ T cells differentiate into different types of effector T cells with distinct functions (100, 101). In addition, DCs can activate other immune cells of the innate immune system, such as NK cells and macrophages (102), DCs connect the innate and adaptive arms of the immune system and are essential for the induction of immune responses.

Recent reports have implicated lipid metabolism as an essential regulator of DC development, maturation, and function (103). Excessive lipid accumulation in DCs is a major cause of DC dysfunction. In a clinical study on lung cancer, Arai et al. observed that the fluorescence intensity of lipid accumulation in DCs was significantly higher in patients with stage III/IV lung cancer compared with the controls. The accumulated lipids were mainly TGs, and this dysfunction impaired DC function, contributing to tumor immune escape (104, 105). In a mouse model of ovarian cancer, up-regulation of FASN expression in tumor cells resulted in excessive lipid accumulation, which inhibited the activation of T cells infiltrating the tumor by DCs (106). Kratchmarov et al. found that the inhibition of FAO with the CPT1 inhibitor etomoxir did not affect the overall development of cDCs or pDCs, although it significantly modified their subset frequencies, with an increase in cDC2 and a decrease in cDC1 (107, 108). These findings suggest that the subsets of DCs is determined by immune signals and microenvironment, and metabolic reprogramming allows them to function in different microenvironments.

Interestingly, FAO, regulated by signaling through peroxisome proliferator-activated receptors (PPARs), AMP-activated protein kinase (AMPK), mechanistic target of rapamycin (mTOR), signal transducer and activator of transcription 3 (STAT3), and peroxisome proliferator-activated receptor gamma coactivator 1-alpha (PGC-1α), is critical for DC differentiation. For example, PPARs, key transcription factors regulating FAO, are significantly upregulated in granulocyte-macrophage colony-stimulating factor (GM-CSF)- and interleukin-4 (IL-4)-induced moDCs and are essential for their generation (109, 110). AMPK phosphorylation upregulates FAO, inducing an immunosuppressive phenotype in tolerant DCs (111), and the activation of the JAK2-STAT3 signaling pathway has been shown to inhibit dendritic cell (DC) maturation (112). These mechanisms highlight the intricate relationship between lipid metabolism and DC function within the TME. Future investigations should therefore prioritize elucidating how the PPAR, AMPK, mTOR, STAT3, and PGC-1α signaling networks dynamically interact to regulate FAO, thereby orchestrating the metabolic reprogramming that enables distinct DC subsets to adapt and function within the diverse and challenging niches of the TME.

### 3.4 Natural killer cells

NK cells are cytotoxic lymphocytes of the innate immune system, capable of killing tumor cells and virus-infected cells. They exhibit direct effector functions against cellular targets and play a role in generating, forming, and maintaining multicellular immune responses. Glucose metabolism is critical for key NK cell functions, including cytokine production, receptor-mediated activation, ligand expression, degranulation, and the regulation of glycolysis, oxidative phosphorylation (OXPHOS), and adenosine triphosphate (ATP) production (113, 114).

Although NK cells are major players in the innate elimination of viral, bacterial, and tumor cells, research on lipid metabolism in NK cells and tumor-associated NK (TANK) cells remains limited. Nevertheless, the importance and clinical relevance of the crosstalk between lipid metabolism, NK/TANK cells, and tumors have been clearly demonstrated. A growing body of evidence suggests that the TME produces soluble modulators that inhibit NK cell maturation, proliferation, and effector functions. These immunosuppressive factors may directly affect NK cells or indirectly influence them by inducing the production of additional immunosuppressive molecules by other immune cells, such as regulatory T cells (Tregs), antigen-presenting cells (APCs), and myeloid-derived suppressor cells (MDSCs). Metabolism is a critical component of NK cell immunobiology and a key determinant of their response to immunotherapy in tumors.

Therefore, in the future, we should focus on exploring the specific mechanisms by which lipid metabolism regulates NK cell function dynamically and the effects of lipid metabolic heterogeneity in the TME on NK cell function. That is, we should use patient samples from clinical trials for multi-omics profiling in subsequent studies. By integrating single-cell transcriptomics and lipidomics with multi-omics technologies, we can delineate the lipid metabolic signatures underlying NK cell dysfunction and identify potential targets for therapy. Using clinical trials and multi-omics technologies will be crucial for the further development of NK cell-based therapies for cancer with improved therapeutic effect and translational value.

### 3.5 Tumor-associated neutrophils

Neutrophils are differentiated from hematopoietic stem cells in the bone marrow and further mature in the bloodstream. In addition to being fighters against pathogenic infections, neutrophils also serve as coordinators that regulate, target, and activate inflammatory and adaptive immune responses. Neutrophils can express various cytokines and immunosuppressive molecules and also interact with other effector cells, such as B cells, DCs, macrophages, and T cells. Because of their functional plasticity, neutrophils can either kill tumor cells or promote tumor growth. Lipid metabolism regulates the immune function of neutrophils by affecting their activation, migration and effector functions.

In 2009, Friedlander et al. first proposed the classification of neutrophils into anti-tumor (N1) and pro-tumor (N2) subsets (115). Neutrophil activation and function depend on FAs utilization and oxidation. Free FAs enter the FAO pathway and undergo oxidative phosphorylation (OXPHOS) to produce adenosine triphosphate (ATP), which supports neutrophil differentiation, maturation, and function. In the early stages of tumorigenesis, TANs are predominantly of the N1 phenotype, exerting anti-tumor functions through the activation of interleukin-18 (IL-18) and the secretion of interferon-beta (IFN-β). However, as tumors progress, TANs often shift to the N2 phenotype. N2-like TANs promote tumor progression by regulating cancer cell proliferation, angiogenesis, and metastasis. They also inhibit T cell responses and disrupt T cell function and polarization by generating reactive oxygen species (ROS) and overexpressing programmed death ligand 1 (PD-L1) and arginase-1 (116, 117).

Peroxisome proliferator-activated receptor gamma (PPARγ) is a key transcription factor that regulates adipogenesis and stimulates lipid droplet formation. PPARγ ligands not only promote adipogenesis but also induce neutrophil differentiation (118). Similarly, during the differentiation of hematopoietic progenitor cells into neutrophils in the bone marrow, all-trans retinoic acid (ATRA) and granulocyte colony-stimulating factor (G-CSF) stimulate the accumulation of lipid droplets (LDs) in neutrophils via PPARγ (119). Emerging evidence highlights the critical role of neutrophils in tissue damage and repair processes, mediated by lipid metabolism-driven inflammatory responses in chronic inflammatory diseases (120). Consequently, therapeutic strategies aimed at modulating neutrophil lipid metabolism—such as targeting key metabolic pathways or developing precision-based pharmacological agents—hold significant promise. These strategies not only can improve the neutrophil immune functions, but also provide new approaches for cancer and inflammatory diseases therapy.

### 3.6 Myeloid-derived suppressor cells

MDSCs are generated from immature myeloid cells and can be divided into two subsets: polymorphonuclear MDSCs (PMN-MDSCs) and monocytic MDSCs (M-MDSCs). MDSCs are closely involved in tumor progression, metastasis, and therapy resistance via the promotion of Treg proliferation and the secretion of immunosuppressive molecules, including arginase 1 (Arg1), inducible nitric oxide synthase (iNOS), reactive oxygen species (ROS), and prostaglandin E2 (PGE2). Tumor-derived exosomes can exert their onco-suppressive effect by activating and expanding MDSCs through the transfer of microRNAs (miRNAs), interleukins (ILs), transforming growth factor-beta (TGF-β), and PGE2 (121, 122).

Tumor-derived exosomes can exert their onco-suppressive effect by activating and expanding MDSCs through the transfer of microRNAs (miRNAs), interleukins (ILs), transforming growth factor-beta (TGF-β), and PGE2 (121, 122).

Fatty acid transporter protein 2 (FATP2) is responsible for lipid accumulation in PMN-MDSCs. The lipid content is essential for the expression of pro-inflammatory genes and suppression of CD8+ T cells. Pharmacological inhibition of FATP2 can attenuate lipid accumulation, ROS production, and immunosuppressive activity, and further inhibit tumor growth (123). Tumor-infiltrating PMN-MDSCs increase FAs uptake and activate FAO. Inhibition of FAO blocks their immunosuppressive pathways and reduces the production of inhibitory cytokine (124). The serine/threonine kinase PIM1 regulates lipid oxidation through peroxisome proliferator-activated receptor gamma (PPARγ)-mediated activity. Enhanced PPARγ expression rescues the metabolic and functional defects in Pim1-/- PMN-MDSCs (125), underscoring the critical role of lipid metabolism in MDSC-mediated immunosuppression.

### 3.7 Cancer-associated fibroblasts

In addition to immune cells, CAFs are a major stromal component of the TME. CAFs exhibit remarkable heterogeneity and plasticity, playing a crucial role in the TME formation, tumor metabolic reprogramming, proliferation, invasion, and immune modulation. In the TME, CAFs are activated by signaling pathways involving transforming growth factor-beta (TGF-β) and lysophosphatidic acid (LPA) (126, 127). They synthesize and secrete lipids and biologically active lipid signaling molecules. When biologically active lipids, such as lysophosphatidylcholine (LPC), are overexpressed in CAFs, they can be released into the TME and taken up by tumor cells, promoting tumor proliferation and migration through intracellular lipid metabolic reprogramming (128, 129).

CAFs also secrete cytokines or engage in direct cell-to-cell interactions, promoting malignant phenotypes in tumor cells, enhancing therapeutic resistance, and modulating the activity of other tumor-infiltrating immune cells (130). In response to the TME, CAFs reprogram their lipid metabolism by upregulating key enzymes such as FASN and SCD, thereby increasing lipid synthesis, storage, and secretion (128, 131).

## 4 Targeting lipid metabolism in cancer therapy

### 4.1 Immunotherapy and the challenge of resistance

Chemotherapy and immunotherapy represent the cornerstone treatments for metastatic cancer. While standard chemotherapeutic agents include antibiotics, plant-derived compounds, and platinum-based drugs, the most widely utilized immunotherapeutic agents are immune checkpoint inhibitors, such as monoclonal antibodies targeting PD-1, PD-L1, and CTLA-4. The CTLA-4 pathway primarily plays a role in the initial phase of the activation of the immune response. When T cells are activated in the lymph nodes, CTLA-4 competes with the co-stimulatory molecule CD28 for interaction with its ligands on antigen-presenting cells. Since CTLA-4 delivers an inhibitory signal to the T cell, it can serve to suppress the early activation and proliferation of the T cell (132). Tumor cells can use this mechanism to suppress initial activation of the immune system (133). In contrast, the PD-1 pathway primarily plays a role in the effector phase of the immune response, mainly in peripheral tissues and the TME. Under normal conditions, engagement of PD-1 on normal cells by its ligands on the cell surface represents a protective mechanism, or self-tolerance, that prevents T cell-mediated damage to the host (134–136). Many tumor cells express high levels of PD-L1. When Tumor-infiltrating T cells express PD-1 and encounter PD-L1 on tumor cells, they receive a powerful inhibitory signal and become “exhausted.” An exhausted T cell is characterized by its weakened ability to induce cytotoxicity and its failure to kill tumor cells, which leads to tumor immune escape (134–136).

In contrast, clinically, inhibitors targeting the CTLA-4 pathway and PD-1/PD-L1 inhibitors exert their clinical effects by antagonizing the respective receptor and unleashing T cells from their inhibitory states, which restores anti-tumor immunity. Their clinical benefits in NSCLC have been validated and they have been incorporated into clinical practice (134–136). However, among patients, there is still a high degree of heterogeneity in responses to these therapies. One of the major sources of this heterogeneity is the reprogramming of metabolism in the TME, particularly the dysregulation of lipid metabolism, which has been shown to play a critical role in mediating resistance to immunotherapeutic agents.

### 4.2 Mechanisms of lipid metabolism-mediated immunosuppression

Reprogrammed lipid metabolism in the TME represents one of the key mechanisms underlying the immune evasion and resistance to checkpoint inhibitor therapy. This immunosuppression involves the following mechanisms: First, metabolic competition. Cancer cells overexpressing FAs transporters, such as CD36, scavenge extracellular lipids extensively, resulting in cancer cell nutrient deprivation and leading to functional exhaustion of T cells (83, 137). Second, active immunosuppression. Notably, some specific lipid mediators are involved in immunosuppression, including oxysterols and PGE2. These substances are generated by tumor cells and immunosuppressive cells, including Tregs and M2-type TAMs. On one hand, they directly impair the cytotoxicity of CD8+ T cells (138, 139). On the other hand, they upregulate inhibitory receptors, such as PD-1 and TIM-3. These two mechanisms collectively induce the exhausted T cell phenotype (140, 141). Therefore, targeting key nodes in lipid metabolism may conversely enhance T cell function and render cancer immunotherapy more sensitive.

Resistance to immunotherapy involves the following mechanisms of tumor cell intrinsic factors (impaired antigen presentation, upregulation of immune checkpoint molecules, hyperactivated oncogenic signaling pathways); TME (infiltration of immunosuppressive cells, dysregulated cytokine networks, metabolic dysfunction); and host systemic factors (gut microbiota composition, host immune status). Notably, lipid metabolic reprogramming in the TME participates in T cell exhaustion while promoting the survival and function of immunosuppressive population, including Tregs (142). Clinical practice also shows that patients with favorable response to immunotherapy exhibit different gut microbial species composition, while antibiotics treatment may result in reduced efficiency of immunotherapy (143).

### 4.3 Therapeutic strategies targeting lipid metabolism

There are major efforts underway towards the development of therapeutic agents targeting lipid metabolism at multiple nodes; many inhibitors of lipid-metabolizing enzymes are highly promising in large preclinical studies (7, 9, 144). Targeting FA Synthesis. FASN inhibitor TVB-2640 is in phase II clinical trials as monotherapy for non-small cell lung cancer with KRAS mutations (NSCLC, KRAS, NCT03808558), in combination with paclitaxel and trastuzumab for triple-negative breast cancer (NCT03179904), and in combination with the anti-angiogenic drug bevacizumab for high-grade astrocytoma (NCT03032484). Preclinical studies have demonstrated that ACC inhibitors ND-646 and ND-654 significantly inhibit the growth of lung tumors in mice and hepatocellular carcinoma (HCC) in rats, respectively. Additionally, the ACC inhibitor ND-630, initially developed for non-alcoholic steatohepatitis, is undergoing phase I trials for cancer treatment (145). Targeting Cholesterol Synthesis. Statins, a class of cholesterol-lowering drugs that selectively inhibit HMGCR, have gained attention for their anti-tumor properties and are being tested in multiple clinical trials as anticancer agents (146). Statins such as pitavastatin, fluvastatin, and simvastatin have been shown to inhibit tumor cell proliferation and angiogenesis while promoting apoptosis (147–149). Statins suppress cancer cell development by blocking the mevalonate pathway and disrupting the transcriptional responses dependent on Yes-associated protein (YAP) and transcriptional coactivator with PDZ-binding motif (TAZ), key regulators of normal organ growth and tumor progression (54). Additionally, statins modulate lipid rafts by lowering cholesterol levels, influencing tumor development. They can also induce autophagy and ferroptosis and alter the TME to exert anti-tumor effects, although the precise mechanisms remain under investigation (150). To preempt the compensatory surge in exogenous cholesterol uptake triggered by synthesis inhibition, a promising strategy is the concurrent disruption of both pathways. Combining statins with LDLR-targeting agents or efflux inhibitors may thus achieve a more durable suppression of tumor cholesterol metabolism.

### 4.4 Combination therapy: checkpoint inhibitors and metabolic modulators

The strategic combination of immune checkpoint inhibitors with pharmacological modulators of lipid metabolism represents a promising therapeutic frontier. For instance, investigation of the experimental agent MK1775 in lung adenocarcinoma demonstrated its ability to sensitize tumors to PD-1 blockade by reprogramming FAs metabolism and altering interactions between TAMs and CD8+ T cells within the TME (151). Two independent studies found that statins and PD-1 inhibitors were administered in combination in animal models with a synergistic effect, resulting in about 40% tumor regression and reversal of T cell exhaustion (152). Although such synergy seems promising, there are several major challenges to the clinical implementation of combination strategies. Significant metabolic heterogeneity within and between tumors may result in diverse treatment responses. The safety of combination regimens should be rigorously examined, especially the off-target effects of metabolic inhibitors on immune cells. In addition, efficient targeted delivery and maintaining therapeutically effective drug concentrations in tumor tissues are critical for successful treatment.

### 4.5 Future directions: integrating advanced technologies

Advanced technologies should be used in the future to confront the above challenges.

Lipidomics is an interdisciplinary field characterized by the systematic identification and characterization of the complete lipid repertoire in living organisms. The main application value of lipidomics technologies in the research of oncology is that they can accurately delineate tumor metabolic heterogeneity and can identify specific lipid profiles associated with resistance to immunotherapy (153). Research on hepatocellular carcinoma found that a low abundance of polyunsaturated fatty acids and high abundance of specific sphingomyelins in the TME were associated with CD8+ T cell exhaustion and limited the effectiveness of anti-PD-1 treatment (154). These findings offer a preliminary rationale for the use of lipid molecules as predictive biomarkers and interventional targets.

Metabolic Imaging: Metabolic Imaging refers to a class of non-invasive radiological techniques that can dynamically visualize metabolic process in living subjects. Its most prominent advantage is that it can delineate the metabolic state of tumors in both spatial and temporal dimensions. For example, novel single-tracer, multi-parametric PET imaging technology that can simultaneously evaluate tumor blood flow perfusion and glucose metabolic rate in a single examination enables a powerful methodology to identify metabolically aberrant regions and acts as an robust tool to evaluate early treatment response and predict resistance (155).

Nanomaterials: Nanomaterials are mainly used as intelligent drug delivery vehicles in cancer therapeutics. The most important application of nanomaterials is to achieve targeted delivery and controlled release. Compared with conventional drugs, nanomaterials are intelligent drugs that can overcome the limitations of conventional drugs, such as poor solubility, significant systemic toxicity, and low bioavailability. For example, LNPs have been used to deliver peptide-based drugs targeting SREBP or to co-deliver mIL-12 mRNA with PD-L1 inhibitors. These approaches can cause significant reversal of the immunosuppressive microenvironment and overcoming resistance in models of liver and colon cancer (156, 157).

Thus, lipidomics, metabolic imaging, and nanomaterials are fundamentally transforming the landscape of cancer therapeutic research. Overall, these three technical platforms constitute an integrated R&D chain from target discovery to clinical application. However, this innovative paradigm is accompanied by several challenges, such as technical challenges, bottlenecks for clinical translation, and challenges associated with multidimensional data integration.

## 5 Summary and prospect

Lipid metabolism has recently emerged as a major area of interest in cancer biology. The high proliferative rate of tumors and nutrient deprivation/hypoxia induced by tumors result in the fact that lipid metabolism has become the most prevailing metabolic process in cancer cells. There is an intricate and complex interplay between lipid metabolism and the TME. Lipid metabolism in cancer cells is not only regulated by intracellular oncogenic signaling, but also by the TME, which is composed of various cell types, cytokines, and growth factors. Meanwhile, abnormal lipid metabolism can modulate the oncogenic signaling pathways in cancer cells and the neighboring cell populations in the TME. Despite the recent advances in the understanding of the effects of lipid metabolism in cancer immunotherapy, there are still many gaps in understanding the dynamic regulatory mechanisms, TME-driven heterogeneity, and clinical translation. We believe that, in the future, investigations should focus on the following questions: deciphering the complex signaling networks that regulate the program of lipid metabolic reprogramming and defining their precise roles in regulating immune responses are fundamental to resolving the currently existing uncertainties in context-dependent regulation and TME-induced heterogeneity. Meanwhile, efforts should be focused on the development of novel therapeutic strategies that specifically target critical nodes in these metabolic pathways. We believe that targeting lipid metabolism will become a new frontier in cancer immunotherapy with transformative insights and novel therapeutic strategies against cancers as research proceeds.
